# Effects of Frankincense Compounds on Infection, Inflammation, and Oral Health

**DOI:** 10.3390/molecules27134174

**Published:** 2022-06-29

**Authors:** Cássio Luiz Coutinho Almeida-da-Silva, Nallusamy Sivakumar, Homer Asadi, Anna Chang-Chien, M. Walid Qoronfleh, David M. Ojcius, Musthafa Mohamed Essa

**Affiliations:** 1 Department of Biomedical Sciences, Arthur A. Dugoni School of Dentistry, University of the Pacific, 155 Fifth Street, San Francisco, CA 94103, USA; csilva2@pacific.edu (C.L.C.A.-d.-S.); hasadi@pacific.edu (H.A.); 2 Department of Biology, College of Science, Sultan Qaboos University, Muscat 123, Oman; apnsiva@squ.edu.om; 3 Dental Surgery Program, Arthur A. Dugoni School of Dentistry, University of the Pacific, 155 Fifth Street, San Francisco, CA 94103, USA; a_changchien@u.pacific.edu; 4 Research & Policy Department, World Innovation Summit for Health (WISH), Qatar Foundation, Doha 0974, Qatar; walidq3@gmail.com; 5 Department of Food Science and Nutrition, CAMS, Sultan Qaboos University, Muscat 123, Oman; drmdessa@gmail.com

**Keywords:** inflammation, oral health, frankincense, infection, microbiology, immunology

## Abstract

*Boswellia* trees, found throughout the Middle East and parts of Africa and Asia, are the source of frankincense oil. Since antiquity, frankincense has been traded as a precious commodity, but it has also been used for the treatment of chronic disease, inflammation, oral health, and microbial infection. More recently, the bioactive components of *Boswellia* trees have been identified and characterized for their effects on cancer, microbial infection (especially infection by oral pathogens), and inflammation. Most studies have focused on cell lines, but more recent research has also investigated effects in animal models of disease. As natural products are considered to be safer than synthetic drugs, there is growing interest in further developing the use of substances such as frankincense oil for therapeutic treatment.

## 1. Introduction

**Figure 1:**
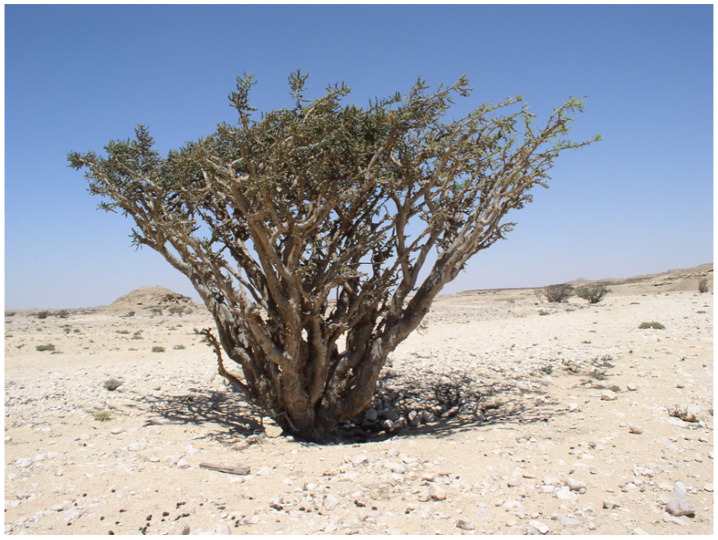
*Boswellia sacra* in Wadi Dawkah, a natural park of frankincense-producing trees in Oman. *Boswellia sacra* photo from Wikimedia.org.

The increasing rate of antibiotics drug resistance, the cost of drugs, and the lack of access to healthcare systems for many people [1,2], especially in less developed countries, support the need for studying traditional medicines for sustainable, cost-effective therapeutic purposes. Frankincense is one of the common sources for traditional medicine [3]. The word “frankincense” is derived from an old French term “franc encens” and means “pure incense” or “pure and noble high-quality incense”. [4] Traditionally, frankincense gum has been burned for the pleasant fragrance it produces. It can be produced as a hardened resin or gum-like material discharged by the *Boswellia sacra* Flück. tree through cuts made in the trunk. Figure 1 shows the *B. sacra* tree, a member of the *Boswellia* genus. Frankincense has been traded and used as one of the most valuable materials since the beginning of written history [5].

The *Boswellia* genus includes around thirty species and is usually found in the arid regions of tropical Africa, the Arabian Peninsula, and South Asia [6], where moisture is supplied by morning mist. For example, *Boswellia serrata* Roxb. is found in India, *Boswellia sacra* in the Arabian Peninsula, *Boswellia papyrifera* (Caill. ex Delile) Hochst. and *Boswellia rivae* Engl. in Ethiopia, *Boswellia neglecta* S.Moore in Eritrea, and *Boswellia carterii* and *Boswellia frereana* Birdw. in North Africa and Somalia [7]. However, frankincense is obtained mostly from *B. frereana*, *B. sacra*, *B. papyrifera*, and *B. serrata*, which grow in Somalia, Yemen, Oman, India, and Pakistan [8]. Although many *Boswellia* species are known to produce frankincense, *B. serrata* (India), *B. sacra* (Oman), and *B. carterii* (Somalia) are the major sources of commercial frankincense [9]. Table 1 includes a comprehensive list of all species of *Boswellia* trees as of 17 June 2022, according to The World Flora Online [10], and their geographical distribution [11].

The oleoresin from the *Boswellia* trees has been considered as a treasure for more than a millennium due to its aromatic and medicinal applications [4]. Frankincense resin is secreted as a milky-white, sticky liquid when the plant is injured, and it is also known as olibanum [12]. Olibanum is produced mainly by four different species of Boswellia, which are *B. serrata, B. carteriii*, *B. frereana*, and *B. sacra* [12,13]. The oleo gum resin produced by members of the genus *Boswellia* helps the plant defend against various infections and pests [14] and has been used in religious ceremonies, perfume production, and as a phytomedicine since antiquity [15]. Recently, the essential oils derived from frankincense are gaining high economic importance internationally for aromatherapy and perfumery [16].

For the present review, the PubMed database, Google Scholar database, Google database, the “Britannica” website, Science Direct, and “The World Flora Online” website were searched using terms that included “frankincense”, “boswellic acids”, and “frankincense and oral pathogens”. Exclusion criteria were non-English-language papers. With the present review, we aim to summarize the most recent research on the effects of frankincense compounds on microbial infection and inflammatory diseases. The effects of frankincense compounds on oral microbial infection are a focus of this review since there is a strong link between systemic and oral health [17].

## 2. Description of Frankincense Resins, Oils, and Boswellic Acids

The resin is available in yellowish, bluish, and greenish shades, and is composed of 3–8% volatile oils containing various terpenes and sesquiterpenes, 60–70% resin, and 27–35% gum [48]. In the mid-twentieth century, it was found that the gum contains two types of polysaccharides [49]. More recently, the composition showed that the monosaccharides present were predominantly galactose, arabinose, and glucuronic acid, along with small amounts of rhamnose and glucose [50]. However, recent scientific advancements allow us to identify the molecular content of diverse frankincense products such as incense mixtures, components of conventional medicines, and archaeological specimens.

The concentrated volatile, aromatic liquid obtained from different parts of the plant such as roots, seeds, leaves, flowers, and resins is called an essential oil. Mostly, a distillation process is used to extract the essential oil from *Boswellia* plants [51]. The main chemical constituents of frankincense oil are pentacyclic triterpenoids [52], tetracyclic triterpenoids [53], and a variety of other oils [54]. The most common characteristic and intensely studied component in frankincense are the pentacyclic triterpenoids [7,55].

**Figure 2:**
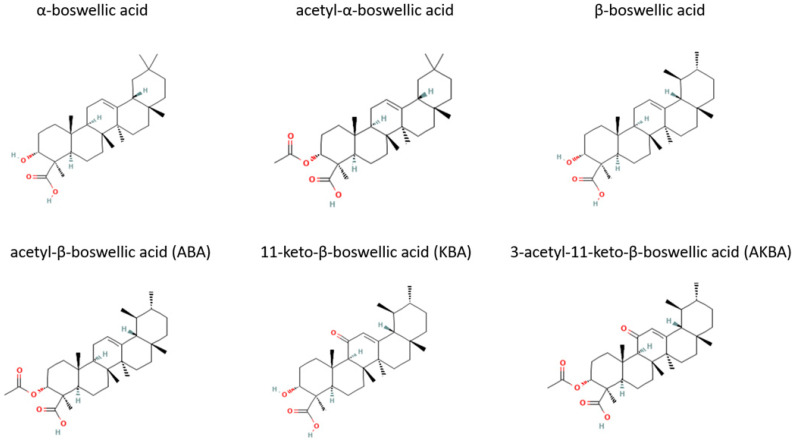
Chemical structure of common boswellic acids found in frankincense. Two-dimensional chemical structures were obtained from Pub Chem. Pub Chem CIDs are as follows: α-boswellic acid, 637234; acetyl-α-boswellic acid, 15181201; β-boswellic acid, 168928; acetyl-β-boswellic acid (ABA), 11386458; 11-keto-β-boswellic acid (KBA), 9847548; 3-acetyl-11-keto-β-boswellic acid (AKBA), 11168203.

The major active components of frankincense are the boswellic acids (BA), which are extracted from the woody parts of different *Boswellia* trees. The representative phytochemicals are β-boswellic acid, acetyl-β-boswellic acid (ABA), 11-keto-β-boswellic acid (KBA), 3-acetyl-11-keto-β-boswellic acid (AKBA), α-boswellic acid, and acetyl-α-boswellic acid. Figure 2 shows the chemical structure of the main BA.

The chemical composition and yield vary according to different factors such as the geographical region of the tree, the wood surface area, the collection season, the collection method, the age of the tree, and the storage conditions [56]. For instance, the gum resin from *B. serrata* has relatively similar quantities of 11-keto-β-boswellic acid (3–4.7%) and acetyl-11-keto-β-boswellic acid (2.2–2.9%) [7]. However, the resin from *B. carterii* contains 0.5% 11-keto-β-boswellic acid and 3.3% acetyl-11-keto-β-boswellic acid [7].

The gum resin of *B. carterii* reported from Somalia has α-thujene (19.2%), sabinene (9.4%), limonene (7.8%), and α-pinene (7.2%) as the main constituents [57]. This is a native plant of the Red Sea region of North-East Africa, where it is abundant in Somalia and Ethiopia. Other studies on the composition of the oleo resin reported different components in different concentrations. For instance, Wahab reported that the oil contains 62.1% esters, 15.4% alcohols, 9.9% monoterpene hydrocarbons, and 7.1% diterpenes [58], and Wang et al. reported octyl acetate (60.0%), octanol (12.7%), and *p*-cymene (8.7%) [59]. The gas chromatography–mass spectrometry (GC/MS) analysis of the hydro-distillate of *B. carterii* resin showed the presence of 40 different constituents. The major constituents were verticiol (14.48%), isobutylcyclopentane (12.25%), *n*-octyl acetate (9.20%), and 9-oxabicyclo-[6.1.0]-non-3-yne (9.12%) [60]. However, the composition of the resin differs due to different factors such as climate, geography, season of the year, and the extraction conditions. This notion is corroborated by a report from Chen et al. (2013) which observed low concentrations of the above components [61], while other studies reported higher concentrations of *n*-octyl acetate [61,62].

*Boswellia dalzielii*, which grows in West Africa, is an understudied species. However, a study conducted using essential oil from 21 oleoresin samples from northern Nigeria and two samples from Ghana revealed that α-pinene was the dominant component (21.7–76.6%), followed by α-thujene (2.0–17.6%), myrcene (up to 35.2%), *p*-cymene (0.3–15.6%), and limonene (1.1–32.9%) [63]. It was reported that out of the 29 compounds obtained from the essential oil from leaves, α-pinene was the dominant component (45.7%) [64]. On the other hand, it was also reported that out of 50 compounds isolated, δ-3-carene (27.7%) was the dominant one [65]. The most abundant chemical components and the type of *Boswellia* plant sources are summarized in Table 2.

## 3. Modern Medicinal Uses of Frankincense

Many plant components from *Boswellia* are used to treat chronic diseases with comparatively few side effects [67,68]. Indian traditional medicine practice narrates that *Boswellia* decoctions were used to treat various gastrointestinal tract conditions such as diarrhea, flatulence, alimentary stoppage, and vomiting [69,70,71,72]. It was believed that the decoctions contain active ingredients from *Boswellia*, which are useful in treating the above-mentioned diseases. Furthermore, the extract was used to treat bronchitis, asthma, hoarseness, dyspnea, cough, and cold [73,74].

The gum extracts or resins from *Boswellia* sp. and their triterpenes, especially BA, have attracted the attention of pharmacologists, medicinal chemists, and biochemists as therapeutic agents due to their efficacy in treating rheumatoid arthritis and chronic inflammation without side effects and toxicity. The gum exudate obtained from the bark of the tree *B. serrata*, also called Indian olibanum, has been extensively used in the treatment of arthritis, asthma, ulcers, and skin diseases by practitioners of Indian traditional medicine [48,58]. It has also been widely used in various formulations for the treatment of inflammation related disorders in the past decade [48,75]. One study analyzed the effects of gum resins of *B. serrata* (900 mg daily divided in three doses for 6 weeks) on human patients suffering from chronic colitis [67,76]. The authors found that out of 20 patients treated with *Boswellia* gum resin, 18 patients showed an improvement in one or more parameters, including stool properties, histopathology and scanning electron microscopy characterization, hemoglobin, serum ion, calcium, phosphorus, proteins, total leukocytes, and eosinophils [67,76]. The control group (10 patients) was given sulfasalazine at a dose of 1 g three times a day for 6 weeks. The authors reported that 6 out of 10 patients in the control group showed similar improvements with the same parameters tested in the *B. serrata* group. This study demonstrates that a gum resin preparation from *B. serrata* might be effective in the treatment of chronic colitis, with minimal side effects [67,76]. The same research group evaluated the effects of *B. serrata* gum resin on the treatment of patients with bronchial asthma [74]. Their data demonstrated that 70% of patients treated with 300 mg thrice daily for 6 weeks showed improvement of disease, evident by the disappearance of physical symptoms and signs such as dyspnea, rhonchi, number of attacks, as well as a decrease in eosinophilic counts. The control group (treated with placebo) only showed 27% of improvements in symptoms [74]. These data imply that gum resin from *B. serrata* could serve as a pharmacological agent in the treatment of bronchial asthma.

Gum resin from *B. serrata* was reported to be a potential remedy for inflammation, and it has been used as a folk medicine for topical and systemic inflammatory diseases for centuries [48]. The oleo gum resin of *B. carterii* has been used in folk medicine to treat cough and asthma and as an embalming fluid for human corpses. In addition, it has been used as an incense, respiratory antiseptic, and diuretic stimulant [58].

The oleo gum resin of *B. serrata* and *B. carterii* were also used in traditional medicine in different countries to treat rheumatic and other inflammatory diseases, including Crohn’s disease and ulcerative colitis [76,77,78]. However, *B. serrata* was reported to be the least effective in recurrent infections [79]. In addition, frankincense extracts and oils were used as antiseptic agents in mouthwash and also used to treat cough and asthma [77]. Furthermore, recent studies on animals and humans have demonstrated the efficacy of resin gum of *B. serrata* to treat inflammatory bowel disease, asthma, osteoarthritis, and rheumatoid arthritis [76,80,81].

**Figure 3:**
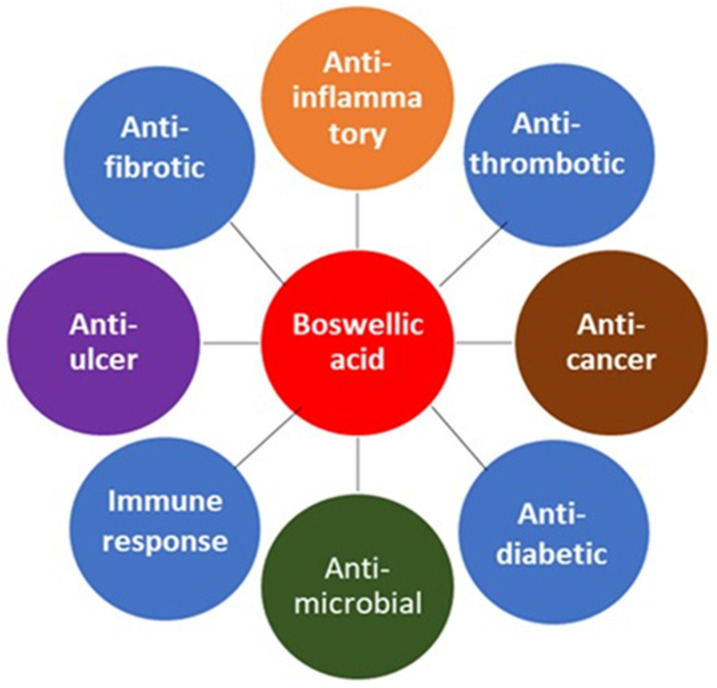
Modern medicinal uses of *Boswellia*.

Though frankincense has been known for its medicinal properties since antiquity, it is still widely used in modern medical treatment. In 2010, Moussaieff and Mechoulam reviewed *Boswellia* resin constituents in vitro, in vivo, and in clinical trials studies [82]. Indeed, in Ayurvedic medicine, different parts of the *Boswellia* tree and derived extracts are used for the treatment of respiratory, gastrointestinal, immune system, and skin conditions. Most of the published work focuses on the pharmacological activities of boswellic acids, specifically on their anti-inflammatory, analgesic, and anti-arthritic properties. As an example, there is growing evidence to support the clinical efficacy of *Boswellia* in osteoarthritis patients. However, there is inadequate evidence to demonstrate the clinical efficacy in rheumatoid arthritis patients [83]. The application of frankincense to treat different diseases of recent times is discussed below and summarized in Figure 3. Frankincense has been used to treat both infections and chronic diseases.

### 3.1. Anti-Cancer

BA is one of the most extensively studied naturally occurring anti-cancer agents due to its anti-carcinogenic, anti-tumor, and anti-hyperlipidemic activities [4,84]. An in vitro study isolated five terpenoids from the gum resin of *B. carterii* and showed that three different compounds exhibited moderate cytotoxic effects against three human cancer cell lines [85]. The isolated compounds (named compounds 1–5) were chemically characterized, and their IC50 values against all three cell lines were described in the study [85]. Among the five compounds, compound 3 showed the following IC50 values: 49.84 ± 3.23 μM (against HepG2 cells, human hepatocellular carcinoma), 57.05 ± 7.85 μM (against A549 cells, human lung carcinoma), and 75.49 ± 7.80 μM (against MCF-7 cells, human breast cancer) [85]. All these values can be compared with IC50 for cisplatin (a chemotherapy drug used to treat several types of tumors): 23.44 ± 3.30 μM (against HepG2 cells), 18.32 ± 3.55 μM (against A549 cells), and 21.42 ± 7.57 μM (against MCF-7 cells) [85]. Even though compound 3 showed promising results, cisplatin was still more effective against the human cancer cell lines. Future studies might determine if the cisplatin and frankincense compounds have synergistic cytotoxic effects against human cancer cells.

AKBA and KBA are the key players involved in the cytotoxic effects of BAs. They inhibit topoisomerase I and IIa, which results in the inhibition of cell growth and proliferation by inducing apoptosis via a caspase-8-dependent pathway in human leukemia, colon, hepatoma, and in various other cancer cell lines in vitro [86,87]. IC50 values for boswellic acid essential oils were calculated and showed that essential oil hydro-distilled at 100 °C produced more potent cytotoxic effects [86]. The IC50 values for T47D cells were 900 and 1450 dilutions for essential oils obtained at 78 °C and 100 °C, respectively. Among the cancer cell lines tested in this study, MCF-7 cells were the most sensitive to essential oils with suppressed cell viability [86]. Boswellic acid acetate inhibited cell growth in vitro in a dose-dependent manner with IC50s of 5.8, 8.7, 7.3, 6.2, 9.8, and 6.6 μg/mL in NB4, SKNO-1, HL-60, U937, K562, and ML-1 cells at 4 days of treatment, respectively [87].

BAs also inhibit protein synthesis by interacting with ribosomal proteins, and thus control cancer development [88]. A study using human colon cancer cells in vitro suggested that *B. serrata* extracts inhibit proliferation, angiogenesis, and migration, and induces apoptosis of the cells by decreasing PGE2 levels [89].

### 3.2. Hypolipidemic and Hypoglycemic Effects

Animal studies have shown that the water-soluble fraction of *Boswellia* is effective in reducing the total cholesterol levels [90]. Extracts of *B. serrata* gum resins can reduce serum cholesterol levels and increase heavy-density lipoproteins (HDL) in rats [90]. In another study, *Boswellia* extracts significantly increased blood HDL levels and, remarkably, decreased blood low-density lipoprotein (LDL) levels, as well as the levels of liver enzymes SGPT and SGOT in patients after six weeks of treatment with *Boswellia* [91]. In contrast, the use of *B. serrata* gum resin for eight weeks at a relatively high dose did not lower glucose and lipid levels in diabetic patients [92].

Several studies have revealed that *Boswellia* gum resin has high hypolipidemic and hypoglycemic effects. Both BA and KBA reduced hyperglycemia and improved biochemical parameters such as the lipid profile. The strong anti-diabetic activity of β-BA and β-KBA was shown by treatments with different concentrations (1, 2, and 10 mg/kg body weight) for 21 days. This treatment significantly improved body weight loss, water consumption, and the concentration of blood glucose levels in diabetic animals [93]. Azemi et al. suggest that the use of appropriate doses of *B. serrata* extracts is vital in obtaining an anti-hyperglycemic effect on blood sugar and also in preventing complications of diabetes [94].

The effect of *Boswellia* resin on metabolic syndromes has been recently reviewed [95]. The extensive literature analysis of animal and human studies revealed that the resin’s protective and therapeutic effects are due to a decrease in hyperglycemia, hyperlipidemia, hypertension, and obesity. The authors refer the readers to this recent review for more details on the proposed mechanisms involved [95].

## 4. Antimicrobial Activity of Frankincense

In general, the bioactive natural compounds produced by different plants are considered better alternatives to synthetic antimicrobial and antioxidant agents. Different compounds obtained from *Boswellia* resins, particularly BA and their derivatives, exhibited diverse biological activities. As considered in this section, the antimicrobial effects can vary depending on the study and the amount of bioactive compounds present in the frankincense extracts.

### 4.1. Activity of Boswellia against Microbes

A study analyzed the antimicrobial and antioxidant activities of methanol extract, ethyl acetate extract, and essential oil from *B. carterii* resin [60]. The methanol extract exhibited the highest antimicrobial activity (MIC of 25 μg/mL) against different Gram-positive bacteria such as *Bacillus subtilis, B. circulans*, and *Streptococcus faecalis*, and a moderate MIC of 400 μg/mL against the Gram-positive *Listeria monocytogenes*, Gram-negative bacteria such as *Escherichia coli* (MIC of 25 μg/mL) and *Pseudomonas aeruginosa* (MIC of 300 μg/mL), and two yeasts, *Candida albicans* (MIC of >1000 μg/mL) and *Saccharomyces cerevisiae* (MIC of 25 μg/mL), compared with essential oil from *B. carterii* resin [60]. The ethyl acetate extract also showed favorable results, with MIC values ranging from 25 to >1000 μg/mL. The IC50 values for the methanol extract, ethyl acetate extract, and essential oils were 5.78, 7.66, and 15.21 mg/mL, respectively [60]. The methanol and ethyl acetate extracts displayed the highest antimicrobial activity against *S. faecalis*, *B. subtilis*, and *B. circulans* [60].

The antimicrobial activity of the essential oils obtained from the hydro distillation of the barks of *B. dioscoridis, B. elongata*, and *B. socotrana* were investigated against two Gram-positive (*Staphylococcus aureus* and *B. subtilis*) and two Gram-negative (*E. coli* and *P. aeruginosa*) bacterial species, as well as against *C. albicans*. The essential oils exhibited different levels of inhibition against the bacterial strains but not against *C. albicans*. The antimicrobial effects of the essential oils showed MIC of approximately 3–17 mg/mL compared with the use of conventional antibiotics (amoxicillin, gentamicin) and antifungals (nystatin) that showed MIC of approximately 3.5–7 μg-mL [96]. In general, Gram-positive strains exhibited higher susceptibility to the essential oils than Gram-negative strains. *B. socotrana* essential oil showed the highest antibacterial activity against *S. aureus* and *B. subtilis* [96].

The oleo gum resin essential oils from *B. carterii* (Somalia), *B. papyrifera* (Ethiopia), *B. serrata* (India), and *B. rivae* (Ethiopia) were individually tested against different fungi and Gram-positive and Gram-negative bacteria. The essential oils exhibited significant antifungal activity against both *C. albicans* and *C. tropicalis*, with the essential oils from *B. carterii* and *B. papyrifera* showing the best activity [97]. The MIC values for *C. albicans* and C. tropicalis were 12.86 and 12.86 μg/mL (*B. serrata*), 6.16 and 6.16 μg/mL (*B. carterii*), 6.09 and 6.09 μg/mL (*B. papyrifera*), and 2.65 and 27.38 μg/mL (*B. rivae*), respectively, compared with the MIC of 0.15 μg/mL for amphoterecin B. The essential oil of *B. rivae* resin exhibited the best activity against *C. albicans* [97]. Limonene present in the essential oils is thought to be responsible for the antifungal activity [98], since oleo gum resin essential oils without limonene lack antifungal activity. The oleo gum resins also showed significant antibacterial effects against Gram-positive *S. aureus* and *S. epidermidis*, with MIC values ranging from 3.52 to 107.20 μg/mL. These values can be compared with the MIC of 1 μg/mL for vancomycin and 2 μg/mL for amikacin used as controls in the experiments [97]. The frankincense compounds against Gram-negative *E. coli* and *P. aeruginosa* showed MIC ranging from 6.60 to 107.18 μg/mL, compared to the vancomycin MIC of 10 and 5 μg/mL for *E. coli* and *P. aeruginosa*, respectively. It is noteworthy that some of the frankincense compounds had antimicrobial effects at concentrations that were comparable or very close to concentrations of the standard antibiotics used in the in vitro experiments. Future studies might confirm and expand the results to in vivo and clinical models.

Oils extracted from the resins of three *B. sacra* cultivars (Najdi, Sahli, and Houjri) were tested for their antimicrobial activities. Grade 1, grade 2, and grade 3 essential oils were prepared from each cultivar. Najdi grade 2 essential oil exhibited antibacterial activity against *S. aureus* and *P. aeruginosa*. The grade 2 essential oils of Sahli and Houjiri were effective against *Propionibacterium acnes* (a dermatological strain). All the essential oils displayed significant antifungal activity in vitro against *C. albicans* and *Malassezia furfur* [99]. Hence, essential oils from *B. sacra* should be further investigated as potential anti-staphylococcal and anti-pseudomonal therapeutic agents.

The antimicrobial activity of *B. carterii* essential oil was investigated against *E. coli*, *P. aeruginosa*, and three strains of *S. aureus*. The oil displayed inhibitory activity against all the pathogens tested. The highest activity was noted against *P. aeruginosa* [97]. However, Chao et al. (2008) reported that *B. carterii* essential oil does not have an inhibitory effect against methicillin-resistant *S. aureus* (MRSA) [100]. Further experiments are needed to resolve the apparent discrepancy.

A study was conducted to investigate the antimicrobial property of essential oils from frankincense and myrrh in independent and combined forms. Essential oils from *B. rivae*, *B. neglecta*, and *B. papyrifera* and myrrh and sweet myrrh, *Commiphora guidotti* and *Commiphora myrrha*, were used in this study. *Cryptococcus neoformans* and *P. aeruginosa* were the most susceptible to oils of both *Boswellia* and *Commiphora* spp, with MIC values ranging between 0.80 and 1.40 mg/mL for *C. neoformans*, and 0.50 and 1.50 mg/mL, respectively [66]. Noteworthy antimicrobial activity was considered for MIC values ≤ 2.00 mg/mL, as the controls with standard antibiotics showed MIC values ranging between 0.2 × 10^−3^ and 0.6 × 10^−3^ mg/mL [66]. The majority (80%) of the oils investigated in this study had significant antimicrobial activity. Different combinations of frankincense and myrrh oils showed that the oils have synergistic properties. Further investigations showed that the most preferable combination against *Bacillus cereus* was *B. papyrifera* and *C. myrrha* [66].

Twenty different frankincense oil samples from various international outlets were analyzed for their chemical components using GC/MS. Though the oils were qualitatively similar, they exhibited quantitative variation. Antimicrobial study was conducted evaluating all the 20 essential oil against five test microorganisms. The antimicrobial effectiveness of the tested oils varied against *S. aureus* depending on the oil sample studied. In this study, it is noteworthy that 6 out of the 20 oil samples tested were considered effective antimicrobials against *B. cereus.* However, moderate to poor activity was observed against *C. albicans* [101].

The effects of *B. sacra* resin, leaf extract, and essential oil on the growth and aflatoxin production by *Aspergillus flavus* and *Aspergillus parasiticus* were studied [102]. Interesting data showed that essential oils from *B. sacra* inhibited the aflatoxin production and the growth of the fungi. However, the resin from *B. sacra* inhibited aflatoxin production but did not impact the growth of the fungi. Nevertheless, the leaf extract of *B. sacra* enhanced aflatoxin production and mycelial dry weight. This study concluded that the resin and essential oil of *B. sacra* could be used as safe preservatives to increase the shelf life of food and feed products.

### 4.2. Activity of Boswellia against Fungal and Bacterial Biofilms

The structured microbial communities attached to a surface are called biofilms. Bacteria, fungi, and protists are capable of forming biofilms. *S. aureus* biofilms are the major cause of device-related infections, which are difficult to treat as the biofilms are generally resistant to conventional antibiotics [103]. Although less frequent, fungal films also develop on implanted medical devices. For instance, *C. albicans*, responsible for causing infections related to indwelling medical devices, is difficult to treat when it forms biofilms because of resistance against most antifungal agents [104,105]. Hence, there is a need to develop new antifungal agents, especially those that target the biofilm stage of the fungi.

*B. papyrifera* and *B. rivae* essential oils were tested against bacterial biofilms. *B. papyrifera* essential oil showed substantial activity against biofilms formed by both *S. epidermidis* and *S. aureus*. The essential oil of *B. papyrifera* also demonstrated noticeable antimicrobial effects on *S. epidermidis* biofilms. Similarly, *B. rivae* essential oil was effective against preformed *C. albicans* biofilms [106]. The essential oils especially inhibited germ tube formation and thus hyphal formation. The hyphal formation is vital for the virulence, biofilm formation, and structural integrity of *C. albicans* [105,107]. A large part of the inhibitory activity of *B. rivae* oleo gum resin oil was shown to be due to the presence of limonene (28%). Hence, further studies are warranted to characterize the antibiofilm activity of other components in the oleo gum resin oil.

### 4.3. Antimicrobial Activity against Oral Pathogens

**Figure 4:**
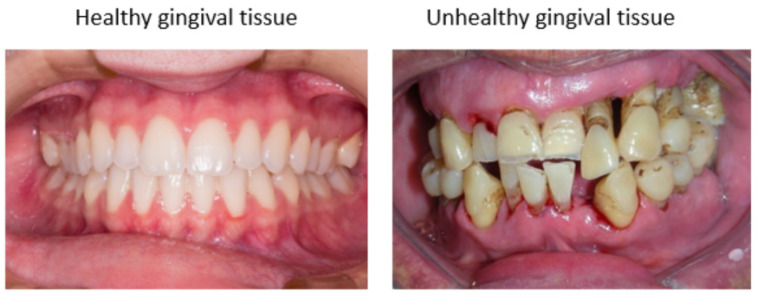
Comparison of healthy and unhealthy gingival tissue. The healthy gingiva is pink in color, while unhealthy gingiva is darker red, and may present bleeding. (Photos from Pixabay.com and Wikipedia.org.)

Dental caries and periodontitis remain two of the major oral diseases that affect more than half of the global population [108]. Periodontitis is often referred as a common inflammatory disease in humans. Figure 4 shows oral cavities with and without periodontitis. The etiology of these diseases has been linked to certain bacterial species, plaque and biofilm formation, and the resultant inflammatory states. Current therapies for treating periodontal disease place an emphasis on biofilm removal. This is exercised via a combination of mechanical (such as scaling and root planing) and antibiotic treatments. However, these treatment modalities are still unable to fully remove biofilms. This has led to an interest in incorporating herbal extracts into current treatment regimens.

Many studies have been performed using the resin of *B. serrata.* As noted above, this resin contains boswellic acid, which are the active components in its anti-inflammatory effects. In addition, both the boswellic acid and the essential oils of *B. serrata* exhibit antimicrobial properties. Of the four major boswellic acids, acetyl-11-keto-β-boswellic acid (AKBA), has consistently demonstrated the greatest antibacterial effects. In fact, several studies have determined that AKBA is the most active component in the resin of *B. serrata* and other *Boswellia* species [109].

In terms of antibacterial effectiveness, AKBA isolated from *B. serrata* demonstrated activity against *Streptococcus mutans, Enterococcus faecalis, Enterococcus faecium, Actinomyces viscosus, Streptococcus sanguinis, Fusobacterium nucleatum, Prevotella intermedia, and Porphyromonas gingivalis*. Furthermore, the post-antibiotic effects of AKBA were determined to be greater than those of ciprofloxacin, and AKBA was also shown to inhibit biofilms of the cariogenic bacteria *S. mutans* [109].

In a double-blinded, randomized clinical trial, the effectiveness of traditional scaling and root planing methods were compared to the use of *B. serrata* extract or powder [110]. The results indicated that the addition of *B. serrata* extract or powder to traditional scaling and root planing treatments leads to better gingival health as measured by gingival, plaque, and bleeding indices, as well as probed pocket depths [110]. The authors concluded that the anti-inflammatory properties of *B. serrata* extract had results comparable or even superior to traditional scaling and root planing treatments.

In another study contrasting the potencies of different forms of frankincense extract, it was shown that hydro-alcoholic extract from *B. serrata* had greater antimicrobial potency than organic extract [111]. While the hydro-alcoholic extract could successfully inhibit *C. albicans, S. mutans, C. krusei,* and *C. glabrata,* organic extract could only inhibit *C. glabrata* at a higher concentration than was needed for the hydro-alcoholic extract.

Current periodontal therapies have often utilized synthetic chemical agents, most notably chlorohexidine, as adjunct to mechanical debridement. However, chronic use of such chemical agents can lead to undesirable side effects, including toxicity to connective tissue, staining, dysgeusia, dry mouth, and allergies [112]. Vahabi et al. analyzed the effects of *B.* serrata, *L. inermis,* and *M. sylvestris* on *Aggregatibacter actinomycetemcomitans,* one of the major species implicated in aggressive periodontitis, as possible alternatives to chlorhexidine. Of the three plant extracts, *B. serrata* had the strongest antimicrobial effect [112]. However, its effects were still inferior to those of chlorohexidine and doxycycline. The antimicrobial effects of frankincense against oral pathogens are summarized in Table 3. According to the data summarized in Table 3, AKBA showed stronger antimicrobial effects against oral pathogens (*P. gingivalis, P. intermedia, E. faecalis, S. mutans, E. faecium, A. viscosus, S. sanguinis*). Therefore, future studies should further examine the antimicrobial effects of AKBA on oral pathogens. Given its antibacterial, antifungal, and antioxidant properties, it is not surprising that frankincense also has promising beneficial effects on oral health.

There is growing evidence that BA is a multitarget agent, since it can modulate several molecular targets, such as enzymes, growth factors, kinases, transcription factors, receptors, and other targets related to survival and proliferation of cells [68]. However, to the best of our knowledge, it remains to be determined whether BA affects molecular targets specific to oral tissues that could affect oral microbial infection and diseases. Some of the molecular targets involved in anti-inflammatory diseases affected by BA will be discussed in the next section.

## 5. Anti-Inflammatory Effects of Frankincense

Many patients consuming non-steroidal anti-inflammatory drugs (NSAID) suffer from adverse effects of the drugs on the gastrointestinal or cardiovascular systems. Many efforts have been made to develop drugs that avoid these complications, but the goal is still far from being achieved. Hence, it is necessary to search for novel, alternative drugs. In this context, *B. serrata* and other *Boswellia* species have gained prominence as potential alternatives as anti-inflammatory herbal remedies. The anti-inflammatory properties of boswellic acids have been well-established by several studies with cells in vitro, and preclinical, and clinical trials [4,7].

Studies on animals and humans have shown the anti-inflammatory potential of *B. serrata* gum resin to treat different inflammatory disorders such as inflammatory bowel disease, rheumatoid arthritis, osteoarthritis, and asthma [80]. Corticosteroids are essential to control cerebral edema but they also cause severe negative impacts. In contrast, the gum resin of *B. serrata* was found to control the peritumoral brain edema associated with glioma [114].

The anti-inflammatory activity of *B. serrata* is due to the presence of α- and β-boswellic acid and other pentacyclic triterpenic acids. These compounds inhibit pro-inflammatory processes by acting on 5-lipooxygenase and cyclo-oxygenase and on the complement system [73,115,116]. Inhibition of prostaglandin synthesis plays only a minimal role in the anti-inflammatory effect of BAs. However, inhibition of 5-lipoxygenase by BA decreases production of leukotrienes, whereas chronic inflammatory diseases are associated with increased leukotriene activity [115].

**Figure 5:**
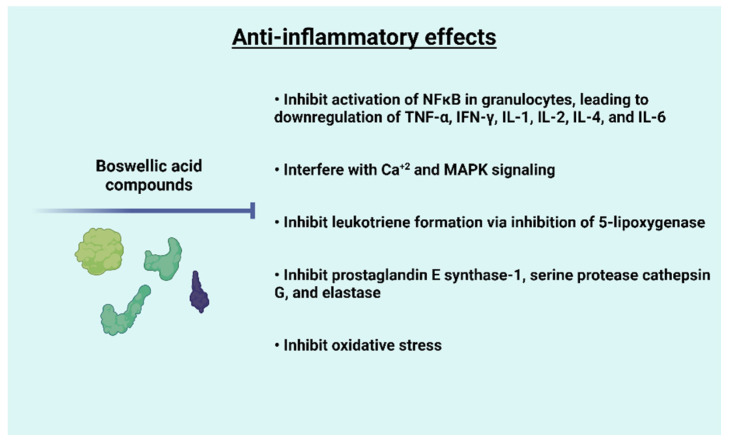
Schematic diagram showing anti-inflammatory effects of boswellic acids and *Boswellia* extracts.

BA is involved in the inhibition of inflammation induced by factors such as histamine, prostaglandins, leukotriene, 5-lipooxygenase, human leukocyte elastase, cytokines, tumor necrosis factor, and free oxygen radicals. Figure 5 summarizes the inhibitory effects induced by BAs. To demonstrate the anti-inflammatory activity of the gum resin of *Boswellia* species, experiments were conducted in animal models. Carrageenan-induced mouse paw edema and rat pleurisy models were employed to test the effectiveness of defined BAs in vivo. This study also demonstrated the suppression of PGE_2_ formation by BAs via interference with mPGES1, which may represent a biochemical basis for the anti-inflammatory effectiveness of BAs [117].

Some limitations of this study must be considered. First, more studies are required to elucidate the antimicrobial and anti-inflammatory mechanisms exerted by frankincense extracts and compounds. Secondly, more studies, including pre-clinical and randomized clinical trials, are needed to better understand the beneficial effects of frankincense extracts and compounds on human health. For more details on the pre-clinical and clinical activities of frankincense, we refer the readers to the recent reviews by Efferth and Oesch [4] and by Hussain et al. [118].

To the best of our knowledge, some limitations to large-scale application of frankincense compounds for clinical studies could be the lack of efficiency in the synthetic methodology for industrial production, and the sustainability of growing enough *Boswellia* trees [118]. According to Groenendijk et al. [119], over the next five decades, the population of *B. papyrifera* trees will decrease to about 10% of its present population due to over-exploitation in harvesting the tree [118].

## 6. Concluding Statement

*Boswellia* trees and natural products derived from the trees have been used since antiquity for the treatment of chronic disease, inflammation, and infection. The identification of the bioactive components of frankincense will allow the characterization of the molecular and cellular basis for their therapeutic effects, and the incorporation of these compounds into products that could be marketed for use in treatment for cancer and inflammation and improvement of oral health.

This review highlights the chemical compounds (boswellic acids) isolated from *Boswellia* gum resins and their potential antimicrobial, anti-tumor, and anti-inflammatory effects. The beneficial role of *Boswellia* gum resins in treating chronic diseases, such as colitis and bronchial asthma, in small studies including human patients, demonstrate the potential of frankincense for treatment in the clinical setting. However, more studies including a higher number of subjects and clinical trials are needed to confirm and expand our knowledge of the use of frankincense for treatment of chronic diseases.

Furthermore, this review discussed the antimicrobial effects of several active compounds of frankincense. Given the high cost of antibiotic therapy and the increase in antibiotic resistance, more research is needed on the potential antimicrobial effects of natural products. We focused on the most recent discoveries of the beneficial effects of frankincense compounds against oral pathogens, given the important connection between oral and systemic health [17].

Gum resin of *Boswellia* is included in the list of substances Generally Recognized As Safe (GRAS), which allows its use as a food additive by the U.S. FDA [109]. One could easily envision the incorporation of frankincense and some of its components such as BAs into mouthwashes and toothpaste, and into topical ointments for inflammatory disorders of the skin.

## Figures and Tables

**Table 1:** List of the current scientific name, official tree name, synonyms, geographical distribution, and references according to the “The World Flora Online website”.

Current Scientific Name	Official Tree Name	Synonyms	Geographical Distribution	References
*Boswellia sacra*	*Boswellia sacra* Flueck.	*Boswellia bhaw-dajiana* Birdw. *Boswellia bhaw-dajiana* var. *serrulata* Engl. *Boswellia carteri* Birdw. *Boswellia carteri* var. *Subintegra* Engl. *Boswellia carteri* var. *Undulatocrenata* Engl. *Boswellia undulatocrenata* (Engl.) Engl.	Arabian Peninsula (Oman, Yemen)	[18]
*Boswellia serrata*	*Boswellia serrata* Roxb. Ex Colebr.	*Boswellia balsamifera* Spreng. *Boswellia glabra* Roxb. *Boswellia thurifera* Roxb. Ex Fleming *Chloroxylon dupada* Buch.-Ham.	India	[19]
-	*Boswellia odorata* Hutch.	-	Niger, northern Nigeria, and eastern Cameron	[20]
-	*Boswellia popoviana* Hepper	-	Socotra	[21]
-	*Boswellia ruspoliana* Engl.	-	Ethiopia, Somalia, Kenya	[22]
*Boswellia socotrana*	*Boswellia socotrana* Balf.f.	-	Socotra	[23]
-	*Boswellia holstii* Engl.	-	Ethiopia, Somalia, Kenya	[24]
-	*Boswellia microphylla* Chiov.	-	Ethiopia, Somalia, Kenya	[25]
-	*Boswellia multifoliolata* Engl.	-	Ethiopia, Somalia, Kenya	[26]
*Boswellia nana*	*Boswellia nana* Hepper	-	Socotra	[27]
*Boswellia ogadensis*	*Boswellia ogadensis* Vollesen	-	Ethiopia	[28]
*Boswellia ovalifoliolata*	*Boswellia ovalifoliolata* N.P.Balakr. & A.N.Henry	-	India	[29]
*Boswellia pirottae*	*Boswellia pirottae* Chiov.	-	Ethiopia	[30]
*Boswellia ameero*	*Boswellia ameero* Balf.f.	-	Socotra	[31]
-	*Boswellia boranensis* Engl.	-	Ethiopia, Somalia, Kenya	[32]
-	*Boswellia bricchettii* (Chiov.) Chiov.	-		[33]
-	*Boswellia bullata* Thulin	-	Socotra	[34]
-	*Boswellia chariensis* Guillaumin	-	Ethiopia, Eritrea, Sudan	[35]
-	*Boswellia dioscoridis* Thulin	-	Socotra	[36]
*Boswellia elongata*	*Boswellia elongata* Balf.f.	-	Socotra	[37]
*Boswellia frereana*	*Boswellia frereana* Birdw.	-	Somalia	[38]
-	*Boswellia globosa* Thulin	-	Somalia	[39]
*Boswellia dalzielii*	*Boswellia dalzielii* Hutch.	-	Northern Nigeria	[40]
*Ambilobea madagascariensis*	*Ambilobea madagascariensis* (Capuron) Thulin, Beier & Razafim.	*Boswellia madagascariensis* Capuron	Madagascar	[41]
*Garuga floribunda*	*Garuga floribunda* Decne.	*Boswellia javanica* Turcz. *Bursera javanica* (Turcz.) Baill. *Garuga abilo* (Blanco) Merr. *Garuga clarkii* Merr. *Garuga littoralis* Merr. *Garuga mollis* Turcz. *Garuga pacifica* Burkill *Garuga pinnata* var. *mollis* Kurz	Southern China, Bhutan, Bangladesh, India, through southeast Asia to the Pacific Islands	[42]
*Boswellia papyrifera*	*Boswellia papyrifera* (Caill. Ex Delile) Hochst.	*Amyris papyrifera* Caill. ex Delile *Boswellia occidentalis* Engl.	Ethiopia	[43]
*Boswellia rivae*	*Boswellia rivae* Engl.	-	Ethiopia	[44]
Boswellia hildebrandtii	*Boswellia hildebrandtii* Engl.	-	Ethiopia, Somalia, Kenya	[45]
-	*Boswellia elegans* Engl.	-	Ethiopia, Somalia, Kenya	[46]
*Boswellia neglecta*	*Boswellia neglecta* S.Moore.	-	Eritrea	[47]

WFO: The World Flora Online.

**Table 2:** Prominent features of compounds in frankincense resin and oils.

Frankincense Type	Country of Origin	Source	Chemical/Active Component	References
*Boswellia dalzielii*	Chad, Mali, Nigeria	Hydro-distilled leaf essential oil	δ-3-carene (27.7%), α-pinene (15.2%), *p*-cymene (9.5%), β-phellandrene (8.5%), isolongifolene (6.2%), and myrcene (5.7%).	[65]
*Boswellia dalzielii*	Nigeria	Hydro-distilled leaf essential oil	α-pinene (45.7%) and α-terpinene (11.5%), trans-sabinene hydrate (4.6%), cis-p-menth-2-en1-ol (2.9%), α-campholenal (2.7%), caryophyllene oxide, and α-phellandrene (2.3%)	[64]
*B. carterii*	Somalia	Gum resin	Esters (62.1%), 1-octyl acetate being predominant (60.0%). Alcohols amount to 15.4%, 1-octanol being the major component (12.7%), and diterpene constituents amount to 7.1%, including cembrene (1.4%), isocembrene (1.8%), incensole (2.7%), and isoincensole (0.8) and a mixture of monoterpene hydrocarbons which amounts to 9.9%.	[58]
*B. rivae*	Ethiopia	Essential oil from frankincense	α-Pinene (36.1–67.7%), δ -3-carene (12.2%), and limonene (12.0%).	[66]
*B. neglecta*	Ethiopia	Essential oil from frankincense	α-Pinene (36.1–67.7%), terpinen-4-ol (11.3%).	[66]

**Table 3:** Boswellic acid extracts or chemical constituents with antimicrobial activity against oral pathogens in vitro.

Microbe	Extract or Chemical Constituent	MIC (μg/mL)	References
*Aggregatibacter actinomycetemcomitans* ATCC 33384 (Gram-negative)	*Boswellia serrata*	512	[113]
*Aggregatibacter actinomycetemcomitans* JP2 NOV99 (Gram-negative)	HAE of *Boswellia serrata*	78	[112]
*Streptococcus mutans* ATCC 25175 (Gram-positive)	KBA AKBA BA ABA	16 2 32 >128	[109]
*Streptococcus mutans* PTCC 1688 (Gram-positive)	HAE of *Boswellia serrata*	50,000	[111]
*Enterococcus faecalis* ATCC 29212 (Gram-positive)	KBA AKBA BA ABA	16 4 8 >128	[109]
*Enterococcus faecium* ATCC 8042 (Gram-positive)	KBA AKBA BA ABA	16 4 8 >128	[109]
*Actinomyces viscosus* ATCC 15987 (Gram-positive)	KBA AKBA BA ABA	8 2 64 >128	[109]
*Streptococcus sanguinis* ATCC 10556 (Gram-positive)	KBA AKBA BA ABA	8 2 128 >128	[109]
*Fusobacterium nucleatum* ATCC 10953 (Gram-negative)	KBA AKBA BA ABA	>128 >128 >128 >128	[109]
*Prevotella intermedia* ATCC 25611 (Gram-negative)	KBA AKBA BA ABA	16 4 32 >128	[109]
*Porphyromonas gingivalis* ATCC 33277 (Gram-negative)	KBA AKBA BA ABA	8 4 32 >128	[109]
*Candida albicans* PTCC 5027	HAE of *Boswellia serrata*	50,000	[111]

MIC: minimum inhibitory concentration; KBA: 11-keto-β-boswellic acid; AKBA: acetyl-11-keto-β-boswellic acid; BA: β-boswellic acid; ABA: acetyl-β-boswellic acid; HAE: hydro-alcoholic extract.

## Data Availability

Not applicable.
